# The role of dopamine signaling in epileptogenesis

**DOI:** 10.3389/fncel.2013.00157

**Published:** 2013-09-17

**Authors:** Yuri Bozzi, Emiliana Borrelli

**Affiliations:** ^1^Laboratory of Molecular Neuropathology, Centre for Integrative Biology, University of TrentoTrento, Italy; ^2^Neuroscience Institute, National Research CouncilPisa, Italy; ^3^Department of Microbiology and Molecular Genetics, University of California IrvineIrvine, CA, USA

**Keywords:** dopamine receptor, seizure, limbic system, temporal lobe epilepsy

## Abstract

Clinical and experimental studies implicate most neuromodulatory systems in epileptogenesis. The dopaminergic system has a seizure-modulating effect that crucially depends on the different subtypes of dopamine (DA) receptors involved and the brain regions in which they are activated. Specifically, DA plays a major role in the control of seizures arising in the limbic system. Studies performed in a wide variety of animal models contributed to illustrate the opposite actions of D1-like and D2-like receptor signaling in limbic epileptogenesis. Indeed, signaling from D1-like receptors is generally pro-epileptogenic, whereas D2-like receptor signaling exerts an anti-epileptogenic effect. However, this view might appear quite simplistic as the complex neuromodulatory action of DA in the control of epileptogenesis likely requires a physiological balance in the activation of circuits modulated by these two major DA receptor subtypes, which determines the response to seizure-promoting stimuli. Here we will review recent evidences on the identification of molecules activated by DA transduction pathways in the generation and spread of seizures in the limbic system. We will discuss the intracellular signaling pathways triggered by activation of different DA receptors in relation to their role in limbic epileptogenesis, which lead to the activation of neuronal death/survival cascades. A deep understanding of the signaling pathways involved in epileptogenesis is crucial for the identification of novel targets for the treatment of epilepsy.

## INTRODUCTION

Epilepsy is a chronic neurological disorder, characterized by spontaneous and recurrent bursts of neuronal hyperactivity (seizures) generally arising in restricted regions of the brain. Seizures may remain confined to their area of origin (“focal” or “partial” seizures) or spread to the whole cerebral hemispheres (“generalized” seizures). The behavioral outcome of seizures strictly depends on the brain regions that are affected by hyperactivity. Seizures have been traditionally characterized as an imbalance between excitatory (glutamatergic) and inhibitory (GABAergic) transmission. The role of glutamate and GABA in epileptogenesis (i.e., the process by which a normal brain develops epilepsy) has been extensively addressed elsewhere (see for example McNamara et al., 2006; Ben-Ari et al., 2012) and will not be further discussed here. Clinical and experimental studies investigated the role of the major neuromodulatory systems in epilepsy (Kurian et al., 2011). Acetylcholine (Friedman et al., 2007; Steinlein and Bertrand, 2010), serotonin (Bagdy et al., 2007), noradrenaline (Weinshenker and Szot, 2002; Giorgi et al., 2004), and dopamine (DA; Starr, 1993, 1996; Bozzi et al., 2011) are all known to regulate seizure activity. In this review, we will focus on the role of DA in seizure onset and spread discussing evidence obtained in human and animal studies. We present a unifying hypothesis on the intracellular signaling cascades triggered by DA and involved in long-term epileptogenesis.

Molecules that stimulate the dopaminergic (DAergic) system such as apomorphine, amphetamines, L-DOPA (L-3,4-dihydroxyphenylalanine), and anti-parkinsonian drugs (e.g., pergolide and bromocriptine) have anti-epileptic action and anti-convulsant effects. Seizures involving the limbic system appear to be the most critically affected by modulation of DA signaling. Brain areas receiving afferents from the mesolimbic DAergic pathway express different types of DA receptors (Bozzi and Borrelli, 2006; Bozzi et al., 2011). Interestingly, while pharmacological studies using animal models support the anti-convulsant effects of DA on limbic seizures (Starr, 1996; Clinckers et al., 2005), contrasting biochemical evidence has been obtained for the presence of DAergic dysfunctions either in the brain of epileptic patients or in animal models of seizure and epilepsy. This suggests that the involvement of DA in seizure and epilepsy is likely due to a dysfunctional control of DA levels or an alteration in the expression of specific receptors. Indeed, levels of DA and its metabolites markedly vary depending on the type of epilepsy and animal models considered (Starr, 1996). However, it is interesting to note that increased levels of DA (Meurs et al., 2008) as well as increased firing of DA neurons (Cifelli and Grace, 2012) were detected in rodent models of temporal lobe epilepsy (TLE). These findings suggest also that variations of DA levels very likely alter the neuromodulatory action of DA on brain circuits of the limbic system.

For instance, a glutamate–DA interaction has been proposed to explain individual susceptibility to epilepsy in limbic areas (Starr, 1996). According to this hypothesis, paroxysmal activity of the cerebral cortex in the epileptic brain would increase the tonic excitation of DA neurons by glutamate. This would then induce phasic release of DA, possibly leading to downregulation or desensitization of DA receptors and subsequently decreased phasic responses. Indeed, DA exerts a marked inhibitory effect on hippocampal excitability through activation of DA D2 receptors (D2Rs). Anti-psychotics (i.e., DAergic D2-like antagonists) lower seizure threshold in epileptic patients and promote seizures in patients with no previous history of the disease. Conversely, seizure inhibition occurs in patients administered anti-parkinsonian drugs such as pergolide and bromocriptine, which both act by stimulating D2Rs (Starr, 1996). Further observations supported the anti-convulsant effect of a low dose treatment with bromocriptine. According to the Starr’s hypothesis, a low dose of a D2R agonist would act through stimulation of presynaptic D2 autoreceptors leading to decreased DA release, while preventing the downregulation of postsynaptic D2R (Chen, 2006). Based on our results using mice lacking D2R (D2R^-/-^ mice), we postulated that D2R activation might exert a neuroprotective action on hippocampal and DAergic neurons against excitotoxicity (Bozzi et al., 2000; Bozzi and Borrelli, 2006). Conversely, activation of DA D1 receptors (D1Rs) has a proconvulsant effect, lowering seizure threshold (Starr, 1993, 1996). The opposite action of D2R and D1R signaling might also be explained by the glutamate–DA interaction hypothesis for limbic epileptogenesis. Indeed, the activation of D1R in cortical tissue samples obtained from children undergoing epilepsy surgery has been shown to induce glutamate receptor-mediated neuronal hyperexcitability (Cepeda et al., 1999). More recent studies performed in animal models during seizures support these results showing a D1R-mediated activation of glutamatergic neurons (Gangarossa et al., 2011; see also below).

These data clearly point to a prominent role of the DAergic circuits in limbic epileptogenesis. Classical pharmacological studies supporting this view have been extensively reviewed by Starr (1993, 1996), to which the reader is referred for a more detailed description. In the next paragraphs, we will summarize recent human studies in support of this hypothesis. Animal studies will then be discussed to highlight the role of specific DA receptor signaling pathways in seizure onset and spread. We will also describe the mechanisms by which DA receptor signaling may affect neuronal excitability and epileptogenesis in the long-term. The potential importance of DA receptor-based drugs for the treatment of epilepsy will be finally discussed.

## HUMAN STUDIES

Recent studies performed on human epileptic patients (**Table 1**) confirm the role of DA-mediated neurotransmission in epilepsy. The role of DA in epilepsy is most likely mediated by the neuromodulatory effect of this molecule on structures belonging to the basal ganglia and elements of the limbic system. These structures are strongly interconnected and defective DA signaling either in the basal ganglia or in the limbic system might affect the electric properties of neurons located at distal sites through either direct interactions or through feedback mechanisms connecting the cortex to the striatum or other areas. In agreement, it has been postulated that the DAergic transmission in the basal ganglia may provide an inhibitory role (Rektor et al., 2012). Indeed, the basal ganglia are not able to generate specific epileptic activity, as detected by electroencephalographic (EEG) recordings. However, seizures originating in the mesiotemporal lobe of TLE patients can induce EEG changes in the basal ganglia, that may act as filter to the further spread of ictal activity (Rektor et al., 2012).

**Table 1:** Dopamine signaling in epilepsy: human studies.

Epilepsy type	DA target	Analysis/findings	Reference
Ring20	DAT	Reduced [^18^F]-fluoro-L-DOPA binding in basal ganglia	Biraben et al. (2004); Del Sole et al. (2010)
“Absence-like” TLE with sclerosis	DAT	Reduced [^18^F]-fluoro-L-DOPA binding in basal ganglia	Bouilleret et al. (2005)
TLE	DAT	Reduced [^18^F]-fluoro-L-DOPA binding in substantia nigra	Bouilleret et al. (2008)
JME	DAT	Reduced [^11^C]PE2I binding in substantia nigra	Ciumas et al. (2008); Odano et al. (2012)
GTCS	DAT	Reduced [^11^C]PE2I binding in putamen	Ciumas et al. (2010)
ADNFLE	D1R	Reduced [11C]-SCH23390 binding in striatum	Fedi et al. (2008)
TLE with sclerosis	D2R/D3R	Reduced [18F]fallypride binding in hippocampus	Werhahn et al. (2006)
JME	D2R/D3R	Reduced [18F]fallypride binding in putamen	Landvogt et al. (2010)
MTLE	D1R	Increased expression and binding in cortex	Rocha et al. (2012)
MTLE	D2R	Reduced expression in cortex	Rocha et al. (2012)
MTLE	DAT	Increased binding in cortex	Rocha et al. (2012)

*See text for abbreviations.*

### IMAGING

Dopaminergic pathway arising from the ventral mesencephalon [substantia nigra and ventral tegmental area (VTA)] innervate the basal ganglia, the limbic system, and the cerebral cortex (Cooper et al., 1996). Several imaging studies demonstrate that reduced DAergic activity is present in various forms of epilepsy. Reduced [^18^F]-fluoro-L-DOPA uptake (indicating a reduced binding to the DA transporter, DAT) was detected in the basal ganglia of patients suffering of ring20 epilepsy (Biraben et al., 2004; Del Sole et al., 2010), resistant generalized “absence-like” epilepsy and drug-resistant TLE with hippocampal sclerosis (Bouilleret et al., 2005). In TLE patients, [^18^F]-fluoro-L-DOPA uptake was reduced in the caudate, putamen, and substantia nigra (Bouilleret et al., 2008). Reduced DAT has also been detected in patients with juvenile myoclonic epilepsy (Ciumas et al., 2008; Odano et al., 2012) and epilepsy with tonic–clonic seizures only (GTCS; Ciumas et al., 2010).

Alterations of both D1-like and D2-like receptors have been associated to different forms of epilepsy. For example, positron emission tomography (PET) with [^11^C]-SCH23390 revealed a reduced striatal D1R binding in patients with autosomal dominant nocturnal frontal lobe epilepsy (ADNFLE; Fedi et al., 2008), suggesting that neurotransmitter alterations in nigrostriatal DA circuits may contribute to nocturnal paroxysmal motor activity in ADNFLE. A reduced D2R/D3R density (as evaluated by PET using the high-affinity DA D2R/D3R ligand [^18^F]fallypride) was instead found in the temporal lobe of TLE with hippocampal sclerosis. Interestingly, the reduction of [^18^F]fallypride binding did not correlate with hippocampal atrophy, indicating that reduced D2R/D3R density is not just a consequence of the degenerative process, but might play a specific role in the pathophysiology of mesial TLE (Werhahn et al., 2006). The same authors also detected a reduction in D2R/D3R binding in the putamen of patients with juvenile myoclonic epilepsy (Landvogt et al., 2010). A recent study evaluated the expression and binding of both D1R and D2R cerebral cortex samples from surgically treated patients with TLE associated with mesial sclerosis (MTLE). As compared to control samples, higher D1R expression and binding and decreased D2R expression were detected in the neocortex of MTLE patients, whereas D2R binding was unaffected. MTLE samples also presented elevated DAT binding and low tissue content of DA (Rocha et al., 2012). It is interesting to note that in this study, D1R binding negatively correlated with seizure onset age and frequency, and positively with epilepsy duration; conversely, D2R binding positively correlated with seizure onset age and negatively with epilepsy duration (Rocha et al., 2012). These results are in agreement with several data from animal models of TLE (see below), respectively, showing a pro-epileptic and anti-epileptic role of D1R and D2R, and confirm that an altered function of the DAergic system might contribute to TLE.

### GENETICS

Despite the notion that several genetic factors are predisposing to epilepsy, little evidence is available in favor of a direct link between epilepsy and variation of genes coding for protein involved in DAergic neurotransmission. Two studies reported the association of DNA polymorphisms in the DAT gene and idiopathic absence epilepsy (Sander et al., 2000) and alcohol-withdrawal seizures (Gorwood et al., 2003), indicating that genetic variation of the DAT gene may modulate neuronal network excitability and contribute to epileptogenesis. More recently, the genetic variation in DAergic function has been associated with the risk of adverse effect of anti-epileptic drug treatment. Specifically, chronic epileptic patients carrying genetic variants associated with decreased DAergic activity (DA-β-hydroxylase, DBH; catechol-*O*-methyltransferase, COMT and D2R) showed a higher susceptibility to adverse psychotropic effects of levetiracetam (Wood, 2012; Helmstaedter et al., 2013). This suggests that reduced DAergic transmission in epileptic patients might contribute to worsen the outcome of specific anti-epileptic medications.

## ANIMAL STUDIES

**FIGURE 1:**
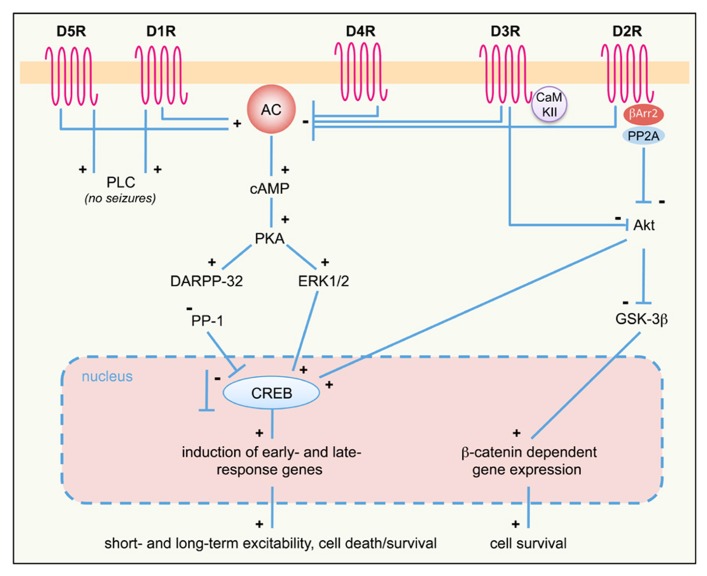
**DA receptor signaling pathways activated in response to seizures.** D1-like (D1R, D5R) and D2-like (D2R; D3R and D4R) DA receptor differently regulate the AC–PKA–ERK pathway. ERK-regulated gene transcription modulates both short- and long-term responses, including neuronal excitability, survival, and cell death. PLC and Akt pathways are also regulated by D1R/D5R and D2R/D3R, respectively. The proposed scheme is a general (though not complete) summary of the intracellular pathways induced by seizures in the limbic system, where all DA receptor subtypes are expressed. Differences may occur, however, in different limbic areas, due to different expression levels of specific DA receptors. See text for details. +, activation; -, inhibition; AC, adenylyl cyclase; βArr, β-arrestin; cAMP, cyclic AMP; CaMKII, Ca^2+^/calmodulin-dependent kinase II; CREB, cAMP response element-binding protein DARPP-32, dopamine and cAMP-regulated phosphoprotein of 32 kDa; D1-5R, dopamine receptors (D1 to D5 subtypes); ERK, extracellular-regulated kinase; GSK-3β, glycogen synthase kinase 3β; PKA, protein kinase A; PLC, phospholipase C; PP-1, protein phosphatase 1; PP2A, protein-phosphatase 2A.

Pharmacological studies demonstrated that the activation of different DA receptor subtypes plays distinct roles in the onset and spread of limbic seizures (Starr, 1996). DA acts through two different types of G-protein-coupled receptors (GPCRs), named D1-like and D2-like (Beaulieu and Gainetdinov, 2011). Activation of D1-like (D1 and D5) receptors results in reduction of seizure threshold and increased seizure severity (DeNinno et al., 1991; Starr and Starr, 1993). Conversely, the effect of D2-like (including D2, D3, and D4) receptors on seizure modulation is mainly inhibitory. Administration of D2-like receptor agonists lowers seizure activity, whereas blockade of these receptors has proconvulsant effects (Starr, 1993, 1996). More recently, studies performed on DA receptor knockout mice (**Table 2**) and the use of compounds acting on specific DA receptor subtypes contributed to dissect the intracellular pathways activated by different DA receptors in response to seizures (**Figure 1**).

**Table 2:** Dopamine signaling in epilepsy: knockout mouse studies.

Mouse	Seizure model	Phenotype	Reference
D1R^-/-^	SKF38393-induced seizures	No seizures	O’Sullivan et al. (2008)
Mice lacking D1R neurons	None	Spontaneous seizures	Gantois et al. (2007)
DARPP-32^-/-^	SKF38393-induced seizures	No seizures	O’Sullivan et al. (2008)
		No ERK activation	Gangarossa et al. (2011)
D5R^-/-^	SKF38393-induced seizures	Increased seizure latency, reduced total EEG seizures	O’Sullivan et al. (2008)
D2R^-/-^	KA seizures	Lower seizure threshold, increased c-fos induction, KA-induced CA3 neuronal apoptosis	Bozzi et al. (2000)
D2R^-/-^	Pilocarpine seizures	Lower seizure threshold	Bozzi and Borrelli (2002)
D2R^-/-^	KA seizures	Increased caspase-3 and GSK-3b activation	Tripathi et al. (2010)
D2R^-/-^	KA seizures	Reduced pAkt(Ser473) in CA3	Dunleavy et al. (2013)
D4R^-/-^	4-Aminopiridine or bicuculline on cortical slices	Increased excitability	Rubinstein et al. (2001)

*See text for abbreviations.*

### D1R

The D1R agonist SKF38393 has a proconvulsant action (Starr, 1996); D1-like receptors (D1R and D5R)-mediated signaling increases cAMP levels and protein kinase A (PKA) activity via the stimulation of adenylyl cyclase (AC) by stimulatory G-proteins (Beaulieu and Gainetdinov, 2011). DA and cAMP-regulated phosphoprotein of 32 kDa (DARPP-32) is a critical downstream target of D1R- and D5R-mediated signaling. PKA-catalyzed phosphorylation activates DARPP-32, and converts it into an inhibitor of protein phosphatase-1 (PP-1). Phosphorylated DARPP-32, by inhibiting PP-1, activates a series of signaling cascades that are important in regulating neuronal excitability (Greengard et al., 1999; Greengard, 2001). In mice, D1-like receptor agonist administration induces seizures and DARPP-32 phosphorylation. Accordingly, seizure behavior is absent or greatly reduced in both D1R and DARPP-32 knockout mice, thus highlighting the crucial role of this signaling pathway in mediating DAergic control of seizures (O’Sullivan et al., 2008). In addition to its direct effect on DARPP-32, D1R-dependent activation of PKA signaling also leads to phosphorylation of extracellular-regulated kinase 1/2 (ERK1/2). Accordingly, seizure-induced ERK activation in the granule cell layer of the dentate gyrus is absent in D1R knockout mice (Gangarossa et al., 2011). Seizures resulting from D1R activation depend on the specific coupling of D1R to the PKA–DARPP-32–ERK pathway. D1-type receptor agonists stimulating the AC pathway increase the levels of Zif268 and Arc/Arg3.1 [two immediate early genes (IEGs) involved in transcriptional regulation and synaptic plasticity] in the dentate gyrus, with a time-course that parallels that of ERK phosphorylation (Gangarossa et al., 2011). Conversely, D1 agonists that stimulate phospholipase C (PLC) but not AC do not induce seizure behaviors (Clifford et al., 1999; O’Sullivan et al., 2008). These results clearly indicate that activation of D1R-dependent signaling has a proconvulsant activity. However, it must be pointed out that postnatal ablation of D1R-expressing striatal neurons results in spontaneous seizures in mice (Gantois et al., 2007), suggesting seizure control may depend on the anatomical integrity of DAergic striatal pathways.

### D5R

D5R activation triggers both cAMP and PLC signaling (Sahu et al., 2009; Beaulieu and Gainetdinov, 2011). Similarly to D1R, D5R-mediated signaling through the cAMP pathway seems to be mainly involved in seizure control. D5R^-/-^ mice treated with the proconvulsant D1R agonist SKF83822, showed an increased latency to first seizure and a reduced total time spent in EEG seizures when compared to wild-type (WT) mice (O’Sullivan et al., 2008). However, it must be pointed out that D5R seems to have less pronounced effects than D1R in regulating synaptic activity (O’Sullivan et al., 2008), as also confirmed by other studies (Granado et al., 2008).

### D2R

Several pharmacological lines of evidence indicate that D2R is the major DA receptor subtype involved in the anti-epileptogenic action of DA in limbic areas. In accordance with imaging studies performed in epileptic patients (**Table 1**), animal studies confirmed that reduced levels of D2R expression are detected in epileptogenic areas in seizing rodents. For example, D2-like binding sites were reduced in the caudate–putamen (CP) of pilocarpine-treated rats (Yakushev et al., 2010) and genetically epileptic GAERS (genetic absence epilepsy rat from Strasbourg; Jones et al., 2010) and WAG/Rij (Wistar Albino Glaxo rats from Rijswijk; Birioukova et al., 2005) rats. Interestingly, WAG/Rij rats also showed a reduced D2-like binding in the CA3 region, confirming a prominent role of D2R signaling in limbic epileptogenesis (Birioukova et al., 2005). The crucial role of D2R signaling in the prevention of hippocampal epileptogenesis is highlighted by the observation that intra-hippocampal administration of remoxipride (a selective D2R antagonist) completely abolished the protective effects of DA against limbic seizures induced by pilocarpine in adult rats (Clinckers et al., 2004).

#### D2R-mediated cAMP-dependent “canonical” pathway

**FIGURE 2:**
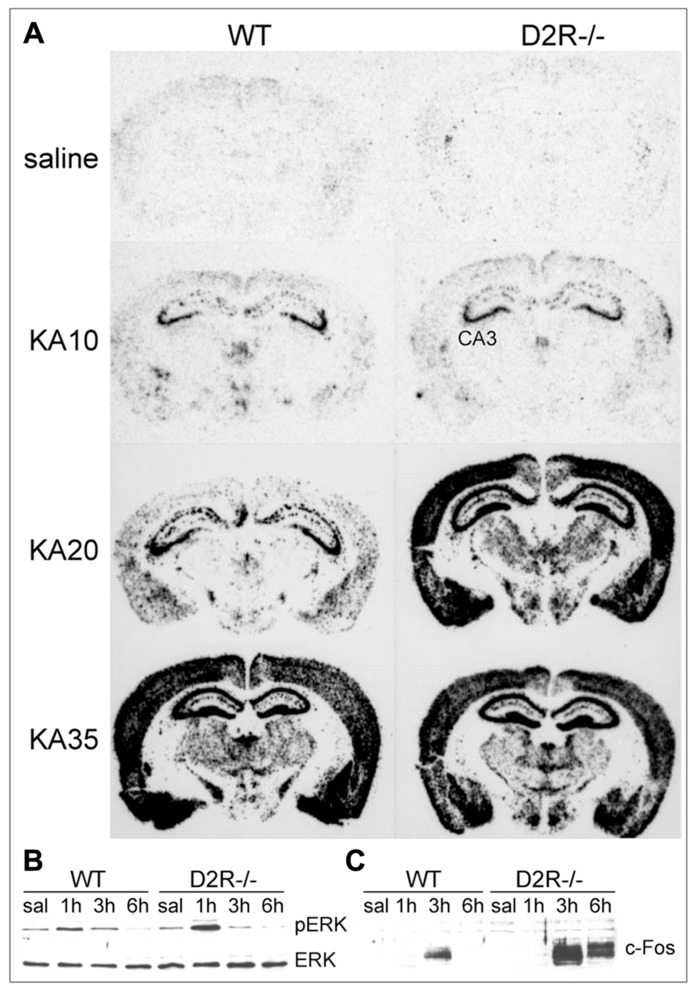
**D2R signaling pathways activated in response to seizures.** **(A)** Pattern of c-*fos* mRNA induction (dark staining) in the brain of WT and D2R^-/-^ mice treated with 10, 20, and 35 mg/kg kainic acid (KA), as indicated. Brains were dissected 3 h after KA administration and coronal sections were processed by mRNA *in situ *hybridization with a c-*fos* specific anti-sense riboprobe (see Bozzi et al., 2000 for experimental details). Administration of 20 mg/kg KA induced limbic motor seizures in D2R^-/-^ but not WT mice (Bozzi et al., 2000; Tripathi et al., 2010; Dunleavy et al., 2013), whereas generalized seizures were observed in both genotypes at 35 mg/kg KA (Bozzi et al., 2000). **(B,C)** Induction of ERK phosphorylation (pERK) and c-Fos protein synthesis in the hippocampus of WT and D2R^-/-^ mice following KA-induced seizures. Mice received a single systemic dose of KA (20 mg/kg) and ERK/pERK **(B)** and c-Fos **(C)** induction were analyzed by immunoblotting on total hippocampal protein extracts at different times after KA (1, 3, and 6 h, as indicated). CA3, pyramidal cell layer of the hippocampus; D2R^-/-^, D2R knockout mice; KA, kainic acid; (p)ERK, (phosphorylated) extracellular-regulated kinase; saline, saline-treated mice; WT, wild-type mice.

D2-like receptor stimulation has an antagonistic effect to D1-like stimulation. D2-like receptors are coupled to Gi proteins that inhibit AC activity. Gi protein activation following DA binding to D2R leads to a decrease of cAMP production (Beaulieu and Gainetdinov, 2011) and subsequent modulation of PKA/ERK signaling (Bozzi et al., 2011). Accordingly, D2R activation is able to counterbalance DARPP-32 activity (Nishi et al., 1997). In the hippocampal kindling model, an increased activation of Gi protein coupled to D2-like receptors was detected in the hippocampus and other brain areas, as evaluated by increased [^35^S]GTPγS *in situ* binding (Alcantara-Gonzalez et al., 2013). Ligand stimulation of G-protein-coupled receptors results into the activation of the associated G-protein and binding of GTP to the catalytic α-subunit. Measuring the binding of [^35^S]GTPγS (a radiolabeled GTP analog) is therefore considered a reliable tool to quantify GPCR activation (Harrison and Traynor, 2003). Increased activation of Gi signaling downstream of D2R autoreceptors might explain the lower DA release detected in the hippocampus of fully kindled animals, which might facilitate seizures (Alcantara-Gonzalez et al., 2013). Accordingly, hippocampal administration of the D2-like receptor antagonist sulpiride induces enhanced DA release and longer seizure duration in kindled animals (Alcantara-Gonzalez et al., 2013). Genetic inactivation of the D2R gene and the consequent impairment of D2R-mediated signaling results in more severe limbic seizures. D2R^-/-^ mice have an increased susceptibility to seizures induced by kainic acid (KA; Bozzi et al., 2000) and pilocarpine (Bozzi and Borrelli, 2002): D2R^-/-^ mice experience generalized limbic motor seizures at doses that are not convulsant in WT mice. The canonical D2R-mediated signaling pathway negatively regulates ERK activity through reduction of cAMP levels and PKA activity, thereby modulating the expression of cAMP-responsive IEGs (Beaulieu and Gainetdinov, 2011). Accordingly, KA administration in D2R^-/-^ mice induces a massive c-*fos* expression (a typical cAMP-responsive gene; West et al., 2002), at a dose lower than in WT mice (**Figure 2A**). KA-induced c-*fos* mRNA upregulation mainly involves the DG-CA3 hippocampal circuit (Bozzi et al., 2000, and **Figure 2A**), thus indicating that the D2R-mediated seizure control mainly involves this limbic circuit. A more rapid and longer-lasting ERK phosphorylation (consistent with the time-course of c-*fos* mRNA induction; **Figure 2A** and Bozzi et al., 2000) is detectable in the hippocampus of D2R^-/-^ mice, as compared to WT controls (**Figure 2B**). In addition, KA-induced seizures result in a stronger and longer-lasting c-Fos protein upregulation in the D2R^-/-^ hippocampus as compared to WT (**Figure 2C**; see also Bozzi et al., 2000). Taken together, these data confirm the critical role of the D2R cAMP-dependent signaling in mediating the first steps of DAergic control of hippocampal activity during seizures.

#### D2R-mediated cAMP-independent pathway

In addition to their increased susceptibility to KA-induced seizures, D2R^-/-^ mice also display increased susceptibility to KA-induced CA3 hippocampal cell death (Bozzi et al., 2000; Bozzi and Borrelli, 2006). This death occurs by apoptosis, as indicated by Bax (Bozzi et al., 2000) and Caspase-3 (Tripathi et al., 2010) upregulation in the hippocampus of KA-treated D2R^-/-^ mice. We recently investigated the intracellular pathways involved in D2R-mediated control of seizure-induced CA3 hippocampal cell death. Several studies show that D2R may also trigger a cAMP-independent pathway. Activation of this pathway following DA binding to D2R results in the inhibition of Akt activity, by dephosphorylation of the threonine 308 (Thr308) residue, leading to activation of glycogen synthase kinase 3β (GSK-3β; Beaulieu et al., 2005, 2007a). Accordingly, we observed GSK-3β activation in the D2R^-/-^ hippocampus after KA (Tripathi et al., 2010), suggesting that upregulation of GSK-3β activity might contribute to increased susceptibility to seizure-induced cell death observed in these mice. However, GSK-3β upregulation in KA-treated D2R^-/-^ mice was independent of Akt phosphorylation at Thr308 (Tripathi et al., 2010), implicating that alternative pathways might contribute to modulate GSK-3β in the hippocampus during epileptic activity. The p38 mitogen-activated protein kinase (MAPK) and Wnt pathways, which have been implicated as potential alternative pathways in regulating GSK-3β activity (Thornton et al., 2008; Inestrosa and Arenas, 2010), are not affected in KA-treated D2R^-/-^ mice (Dunleavy et al., 2013). We were able to show that following KA, phosphorylation of Akt occurs at the serine 473 residue (Ser473) in the CA3 region of WT but not of D2R^-/-^ mice (Dunleavy et al., 2013; CA3 neuron loss following KA is detected in D2R^-/-^ but not WT mice; Bozzi et al., 2000; Bozzi and Borrelli, 2006). Conversely, a strong induction of Akt (Ser473) phosphorylation after KA was detected in the CA1 subregion (where no neuronal cell loss is detected after KA) of both WT and D2R^-/-^ mice (Dunleavy et al., 2013). We therefore proposed that loss of D2R signaling results in reduced Akt (Ser473) phosphorylation, rendering CA3 neurons more vulnerable to apoptosis. Further investigation is required to fully elucidate the Akt/GSK-3β targets involved in D2R-mediated response to excitotoxicity (see also below).

### D3R

Contrasting results were obtained about the role of D3R signaling in seizure modulation. D3R are mainly expressed in the limbic forebrain (Beaulieu and Gainetdinov, 2011; see also below). However, stimulation of D3R has a minimal inhibitory effect on limbic seizures: intra-accumbens pretreatment with D3 agonists delayed the onset of limbic seizures induced by pilocarpine, without any effect on their frequency and severity. In the same model, D2R agonists exerted an anti-convulsant action (Alam and Starr, 1994). Thus, the protective effect of DA on seizure propagation through the limbic forebrain is predominantly mediated by D2R rather than D3R. It has been proposed that D3R participates in D2R cAMP-independent pathway by enhancing D2R-mediated Akt (Thr308) phosphorylation (Beaulieu et al., 2007b). Signaling cascades downstream of D3R also involve Ca^2+^/calmodulin-dependent protein kinase II (CaMKII), that binds to the N-terminal region of the third intracellular loop of D3R (Liu et al., 2009), as well as ERK (Collo et al., 2012) and CREB (cAMP response element-binding protein; Karasinska et al., 2005) phosphorylation, whose activity is negatively modulated by DR3.

### D4R

As the other members of the D2-like receptor family, D4R have a prominent inhibitory role on neuronal hyperexcitability. The frequency of spontaneous synaptic activity and the frequency and duration of epileptic discharges induced by 4-aminopyridine or bicuculline were increased in cortical slices from D4R^-/-^ mice, as well as in brain slices from WT mice treated with a selective D4R antagonist (Rubinstein et al., 2001). *In vivo*, D4R^-/-^ mice showed a reduction of SKF83822-induced seizures, indicating that D4R interacts with AC-coupled D1R to positively regulate D1R-mediated seizures (O’Sullivan et al., 2006).

## BUILDING A UNIFYING VIEW OF DA SIGNALING IN EPILEPTOGENESIS

The results reported in the previous sections clearly highlight the opposite neuromodulatory role of D1- and D2-like receptors on seizures arising in the limbic system. However, most of (if not all) these studies investigated the role of DA signaling on the modulation of *acute *seizures (such as those occurring during an experimentally induced *status epilepticus*). Few data are available suggesting a direct link between DA signaling and *epileptogenesis*, i.e., the establishment of a chronic epileptic condition following an initial precipitating injury. In the next paragraphs we will present current understanding of how altered DA signaling might contribute to a chronic epileptic condition. In the attempt to build up a common signaling network for DA in epileptogenesis, we will first consider (i) the (co)expression of DA receptors in epileptogenic brain areas and (ii) what we know about DAergic modulation of chronic seizures in appropriate animal models of epileptogenesis. Then we will try to explain how altered DA neurotransmission in epileptogenic brain areas might interfere with intracellular pathways involved in long-term hyperexcitability. Finally, we will propose a novel, testable hypothesis on the role of DA receptor signaling in epileptogenesis.

### EXPRESSION OF DA RECEPTORS IN EPILEPTOGENIC AREAS

All DA receptor subtypes are expressed in epileptogenic brain areas. D1Rs are expressed at high levels in the CP, nucleus accumbens (NAc), substantia nigra pars reticulata (SNr), amygdala, and cerebral cortex, and to a lower level in the hippocampus. D5Rs are expressed in the entorhinal cortex, SNr, and hippocampus mainly in the dentate gyrus). Lower levels of expression are found in the NAc and CP neurons. D2Rs are mainly expressed in the CP, NAc, SN pars compacta (SNc), in the ventral tegmental area, cerebral cortex, amygdala, and hippocampus. D3R expression is mainly restricted to areas of the limbic system (NAc, islands of Calleja), but is also present in the SNc, VTA, hippocampus, and cerebral cortex. Finally, D4R expression in epileptogenic areas is limited to the frontal cortex, amygdala, hippocampus, and SN (Beaulieu and Gainetdinov, 2011).

This brief summary clearly shows that most DA receptor subtypes are present in epileptogenic areas within the limbic system. In these areas, DA receptors are generally expressed in different subsets of neurons, but co-expression of different subtypes has also been detected in restricted neuronal populations. For example, D1R and D2R are generally expressed in distinct subpopulations of striatal medium spiny neurons, but a small percentage (5–15%) of these neurons has been shown to co-express both receptors; similarly, 20–25% of pyramidal neurons of the prefrontal cortex do co-express D1R and D2R (Valjent et al., 2009; Beaulieu and Gainetdinov, 2011). In the hippocampus, D1R mRNA is predominantly expressed in the granule cell layer of dentate gyrus, whereas the protein is localized in the molecular layer (Fremeau et al., 1991). D2R mRNA is also expressed in granule cells of the dentate gyrus, but its expression is also detectable in CA1–CA3 pyramidal layers; D2R protein is instead localized in the hilus and stratum lacunosum moleculare (Martres et al., 1985; Bouthenet et al., 1987). According to the expression profile of DA receptor subtypes, it is therefore likely that the signaling cascades depicted in **Figure 1** may cooperate at least in some, restricted neuronal subpopulations within the limbic system, such as dentate granule cells in the hippocampal formation. Indeed, DA has been shown to markedly regulate neuronal excitability in the dentate gyrus (Hamilton et al., 2010), as well as other limbic regions (Tseng and O’Donnell, 2004; Hammad and Wagner, 2006; Rosenkranz and Johnston, 2006) via D1-like and D2-like signaling pathways.

### INVESTIGATING DOPAMINERGIC MODULATION OF CHRONIC SEIZURES IN ANIMAL MODELS OF EPILEPTOGENESIS

The vast majority of studies demonstrating a DAergic modulation of seizure onset and spread were performed on animal models of acute but not chronic seizures (Starr, 1996). Thus, a direct demonstration of a neuromodulatory effect of DA in epileptogenesis is substantially lacking. However, some important indications may be obtained from the limbic kindling model. Limbic kindling consists in the repeated, subthreshold electrical stimulation of the amygdala or hippocampus, that ultimately leads to the expression of chronic seizures (Morimoto et al., 2004). Kindling has been extensively used for the preclinical evaluation of anti-epileptic drugs; many studies demonstrated that drugs showing anti-epileptic effects against limbic kindling also have an anti-epileptic efficacy in clinical TLE (Morimoto et al., 2004). The effect of DAergic drugs on kindled seizures is well-documented. Non-selective DA agonists (such as amphetamines) have an anti-epileptic action. Interestingly, while the prototypical D1R agonist SKF38393 has no effect in this model, D2R-selective compounds do modify seizure threshold. D2R agonists (lisuride) are protective, whereas D2R blockers (haloperidol) exacerbate kindled seizures (Starr, 1996).

The advantages of the limbic kindling model of epileptogenesis are multiple: a precise, focal activation of specific brain areas; a reliable development of chronic epileptogenesis; and a rapid and consistent pattern of seizure propagation and generalization. However, the kindling procedure is labor intensive, and spontaneous seizures develop only after a very large number of stimulations. For these reasons, DAergic modulation of seizure onset and spread has been more extensively studied in pharmacological models of limbic epileptogenesis, namely seizures induced by the muscarinic agonist pilocarpine and glutamatergic agonist KA. These two drugs induce very similar epileptic activity despite their distinct mechanism of action. Pilocarpine and KA initially provoke signs of focal epilepsy (stereotyped pre-convulsive behaviors), due to the activation of limbic areas (dentate gyrus, hippocampal formation, amygdala, entorhinal cortex). From these areas, epileptic activity rapidly propagates to the whole cerebral cortex, culminating in acute motor seizures and *status epilepticus*. Most importantly, pilocarpine- and KA-induced seizures result in extensive neurodegeneration in specific regions of the brain and may lead to the occurrence of spontaneous chronic seizures in the long-term (Turski et al., 1983, 1984; Ben-Ari, 1985; Leite et al., 2002). Using these models, a clear effect of D1R and D2R signaling on the genesis of limbic seizures has been observed, as described in previous paragraphs. However, several questions remain open. Specifically, do DA drugs (namely, D1R antagonists and D2R agonists) have a disease-modifying effect? Do they reduce or stop the occurrence of chronic seizures? The first issue could be addressed by administering DA compounds *after* pilocarpine- or KA-induced *status epilepticus*, and recording the occurrence of spontaneous chronic seizures. To test the anti-convulsant effect of DA drugs onto chronic seizures, D1R- and D2R-selective compounds should instead be administered during the occurrence of spontaneous seizures in appropriate models of chronic epilepsy (such as that resulting from intrahippocampal administration of KA; Antonucci et al., 2008).

### DA SIGNALING AND EPILEPTOGENESIS: A TESTABLE HYPOTHESIS

**FIGURE 3:**
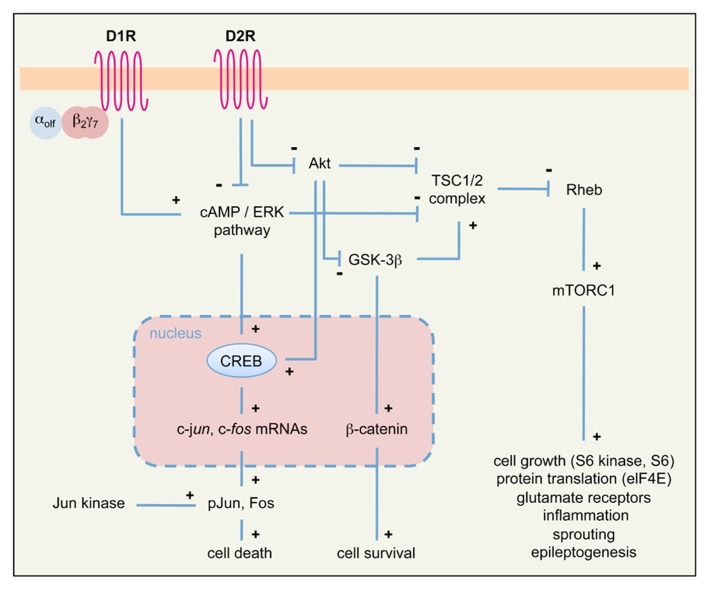
**Simplified diagram of intracellular pathways downstream of DA receptors, potentially involved in seizure-induced cell death and epileptogenesis in the limbic system.** We propose that signaling cascades downstream of D1R and D2R may converge on two principal intracellular pathways (PKA/ERK/Fos/Jun and Akt/GSK-3β/mTOR) to regulate seizure-induced cell death and epileptogenesis. See text for details. α_olf_β_2_γ_7_, trimeric G_olf_ protein; elF4E, elongation factor 4E; mTORC1, mammalian target of rapamycin complex 1; pJun, phosphorylated Jun; Rheb, Ras homolog enriched in brain; TSC1/2, tuberous sclerosis complex 1/2; see also **Figure 2** for other symbols and abbreviations.

Evidence discussed above supports a neuromodulatory role of DA signaling in limbic epileptogenesis. However, the mechanisms by which DA signaling affects neuronal excitability and epileptogenesis in the long-term remain largely unknown. Here we propose that activation of neuronal cell death pathways (a well-known causal factor of limbic epileptogenesis; Bozzi et al., 2011; Henshall and Engel, 2013) following altered DA signaling might contribute to chronic epilepsy. As summarized in **Figures 1** and **3**, stimulation of D1R and blockade of D2R signaling can lead to the activation of neuronal cell death pathways. This phenomenon essentially involves two intracellular cascades: the PKA/ERK/Fos/Jun pathway and the mammalian target of rapamycin (mTOR) pathway.

Canonical, cAMP-dependent signaling through D1R and D2R activates the expression of the IEGs c-*fos* and c-*jun*. Treatment with D1R agonists results in a robust Fos-like immunoreactivity in basal ganglia and limbic structures of rats undergoing pilocarpine-induced generalized seizures (Barone et al., 1993). Recent studies indicate that D1R signaling through the G protein α_olf_β_2_γ_7_ might contribute to seizure-induced neuropathology (Schwindinger et al., 2012). Similarly, D2R receptor blockade by haloperidol induces Fos and Jun B expression during status epilepticus in the hippocampus and striatum (Dragunow et al., 1993); accordingly, KA seizures in D2R^-/-^ mice markedly induce c-*fos* and c-*jun* expression (Bozzi et al., 2000; **Figure 2**). The protein products of c-*fos*/c-*jun* form the AP-1 transcription factor, whose activation regulates the expression of a wide number of cell death genes. The prolonged activation of c-*fos* after acute seizures was proposed as one of the crucial steps that trigger long-term neuronal death (Smeyne et al., 1993; Kasof et al., 1995). Jun phosphorylation (mediated by the c-Jun N-terminal kinase, JNK) activates Jun transcriptional activity and triggers apoptotic neuronal cell death after seizures (Schauwecker, 2000; Bozzi et al., 2000; Spigolon et al., 2010).

D2R signaling also occurs through a cAMP-independent, Akt/GSK-3β-dependent pathway (Beaulieu and Gainetdinov, 2011; see also **Figure 1** and references above). Loss of D2R signaling in D2R^-/-^ mice results in reduced Akt (Ser473) phosphorylation and subsequent overactivity of GSK-3β (Tripathi et al., 2010; Dunleavy et al., 2013), thus rendering CA3 neurons more susceptible to apoptosis. GSK-3β hyperactivity is known to induce hippocampal neurodegeneration (Lucas et al., 2001; Sirerol-Piquer et al., 2011; Llorens-Martín et al., 2013), through mechanisms involving the blockade of the pro-survival β-catenin pathway (De Ferrari and Inestrosa, 2000) as well as the activation of the mTOR pathway (Beaulieu and Gainetdinov, 2011). KA seizures do not alter the β-catenin pathway in the D2R^-/-^ hippocampus (Dunleavy et al., 2013). Thus, it is likely that GSK-3β hyperactivity in KA-treated D2R^-/-^ mice results in the activation of the mTOR pathway. Several evidences support the crucial role of this pathway in epileptogenesis (Cho, 2011; Galanopoulou et al., 2012). For example, the components of the mTOR pathway are upregulated after seizures (Macias et al., 2013) and, most importantly, inhibition of mTOR by rapamycin may ameliorate the development of epilepsy-related pathology and reduce the expression of spontaneous seizures in TLE models (Zeng et al., 2009; Huang et al., 2010). In addition, there is strong evidence that rapamycin may prevent epilepsy and ameliorate its progression in mice lacking the tuberous sclerosis complex genes 1 and 2 (TSC1/2), which act as negative regulators of mTOR (Zeng et al., 2008, 2011). The mechanisms through which mTOR overactivity promotes epileptogenesis and neurodegeneration remain to be understood; according to the multiple action of the mTOR targets, these might involve altered cell growth and morphology, dysregulation of glutamatergic neurotransmission, inflammation, axonal sprouting, and remodeling of epileptogenic circuits (Galanopoulou et al., 2012; see also **Figure 3**).

Taken together, these data lead us to propose that loss of D2R signaling (induced by pharmacological blockade or genetic inactivation of D2R) might contribute to epileptogenesis via the activation of the mTOR pathway. This hypothesis might be tested by checking whether targets of the mTOR complex are upregulated in D2R^-/-^ mice following KA- or pilocarpine-induced seizures. It would be then possible to investigate whether the mTOR inhibitor rapamycin is able to prevent seizures in KA or pilocarpine-treated D2R^-/-^ mice. It is interesting to point out that seizure induction by activation of D1R might also converge onto the mTOR pathway; indeed, this pathway is also activated via PKA/ERK signaling (**Figure 3**), and the D1R agonist SKF81297 was shown to increase the phosphorylation of the mTOR target ribosomal protein S6 in the dentate gyrus, in an ERK-dependent manner (Gangarossa and Valjent, 2012). However, SKF81297 administration did not activate (but rather suppressed) the mTORC1/S6 kinase pathway, suggesting that S6 phosphorylation occurs independently of mTOR (Gangarossa and Valjent, 2012). Further investigation is therefore needed to understand whether stimulation of D1R signaling may promote epileptogenesis via activation of the mTOR pathway.

## EXPLORING THE CLINICAL USE OF DOPAMINERGIC DRUGS IN EPILEPSY

Modulation of limbic seizures by DAergic drugs, as detected in the kindling, pilocarpine and KA models (see above) might predict a similar effect of these drugs on clinical epileptogenesis. However, no known DAergic drug is currently used to treat epilepsy (Beaulieu and Gainetdinov, 2011), despite anti-epileptic effects of DA agonists have been reported in epileptic patients (Starr, 1996). The lack of a systematic investigation of the anti-epileptic efficacy of DA agonists is certainly due to their severe neurological and neuropsychiatric side effects. However, some studies investigated the potential use of the D2R-selective agonist bromocriptine in some forms of epilepsy. Bromocriptine was originally reported to have an anti-epileptic effect in a case of self-induced, drug-resistant epilepsy (Clemens, 1988). Other studies subsequently confirmed that bromocriptine was highly effective in reducing seizure frequency in TLE patients affected by pituitary prolactinomas (Gatterau et al., 1990; Saie and Sills, 2005; Deepak et al., 2007). It is important to observe that these studies did not report severe side effects of prolonged bromocriptine treatment (see also Chen, 2006). Interestingly, in D2R^-/-^ mice, increased seizure susceptibility (Bozzi et al., 2000; Bozzi and Borrelli, 2002) is accompanied by the progressive development of pituitary prolactinomas (Saiardi et al., 1997; Iaccarino et al., 2002; Radl et al., 2013), suggesting that altered D2R signaling might be a common cause of these two conditions. These observations definitely prompt for a better investigation of the anti-epileptogenic efficacy of D2R-selective agonists. Indeed, different D2R agonists (including bromocriptine) have neuroprotective efficacy against KA-induced brain damage (Micale et al., 2006), and recent studies promisingly show that lisuride may reduce seizures occurring after traumatic brain injury (Zweckberger et al., 2010). Further investigation in both animal models and clinical settings is needed to establish the anti-epileptogenic efficacy of D2R agonists.

## CONCLUSION

In this review, we described recent evidence from both human and animal studies supporting the opposite role of D1-like and D2-like receptor signaling in limbic epilepsy. These studies indicate that increased D1R and decreased D2R function might be involved in limbic epilepsy. We propose that altered D1R and D2R signaling might contribute to epileptogenesis via the activation of the neuronal cell death cascades, activated by the PKA/ERK and mTOR pathways. The possible therapeutic application of these findings has been long disregarded, mainly due the severe side effects of DAergic drugs. However, the beneficial effects of selective D2R agonists observed in both animal and human epilepsy would deserve more attention.

## Conflict of Interest Statement

The authors declare that the research was conducted in the absence of any commercial or financial relationships that could be construed as a potential conflict of interest.
